# Prerequisite for reproducible science: a call to embrace code sharing

**DOI:** 10.1016/j.lansea.2024.100472

**Published:** 2024-08-23

**Authors:** Arkaprabha Gun, Tushar Garg

**Affiliations:** a Department of Infectious Disease Epidemiology and Dynamics, London School of Hygiene & Tropical Medicine, London, UK; b Johns Hopkins India, Lucknow, Uttar Pradesh, India; c Department of International Health, Johns Hopkins Bloomberg School of Public Health, Baltimore, USA

At least seven papers in the previous issues of *The Lancet Regional Health — Southeast Asia* have presented analyses derived from India's National Health Family Survey (NFHS), a demographic and household survey with national coverage that is representative at the national, state, and district levels in its latest round.^1^, ^2^, ^3^, ^4^, ^5^, ^6^, ^7^ Conducted by the Ministry of Health and Family Welfare (Government of India) and the International Institute for Population Sciences (a public university), the NFHS is publicly available for research. It is jointly funded by the Government of India and the United States Agency for International Development.^8^ Though the data for these analyses is publicly available, none of the papers have provided the code essential for reproducing their analyses. Analysis of open-access biomedical literature on PubMed Central reveals that articles published in 2020 lacked information on code-sharing (<5%) and data-sharing (<20%).^9^ In contrast, funding and conflict of interest disclosure rates have improved to over 75% over the previous two decades.

The methodological details are insufficient to reproduce the analysis in several papers. For instance, the paper used in our demonstration has no information on applying the complex survey design to generate representative estimates of abdominal obesity.^7^ We, like other analysts, learned in our first independent analysis that the devil is in the details. It is often impossible to reproduce the results without knowing all the steps followed in the analysis, even when a detailed methodology is provided. In our attempt to reproduce the analysis of the paper on abdominal obesity in the Indian population, we highlight the differences in results to demonstrate the necessity of code sharing for both transparency and reproducibility in scientific research.

We analysed obesity using the NFHS-5 (2019–2021) data, applying appropriate survey weights to account for the complex survey design with two-stage sampling. The sample population included adult women (age 18–49 years) and men (age 18–54 years). We excluded adolescents (15–<18 years) for both sexes, pregnant women, and women within 2 months of postpartum because the obesity criteria used did not apply to them. Individuals with missing or out-of-bounds waist circumference or BMI, based on the Demographic and Health Survey (DHS) data dictionary, were also excluded (Supplementary Figure S1). We estimated obesity using abdominal circumference and BMI, and provided national, state, and district-level representative estimates.^10^ We conducted both univariable and multivariable logistic regression using abdominal obesity, age category, type of residence, highest education level, religion, wealth index, caste, and frequency of eating meat — same as the original paper. The analysis was performed in R using the *survey* package with the code publicly available on GitHub.

We included 594,489 women and 86,347 men. The prevalence of abdominal obesity was high in both sexes, though more pronounced in women (Fig. 1). For women, the obesity prevalence was 43.8% (95% CI: 43.5%–44.1%) using waist circumference and 26.3% (95% CI: 26.0%–26.5%) using BMI. For men, the prevalence was 23.4% (95% CI: 22.7%–24.2%) using waist circumference and 25.6% (95% CI: 24.9%–26.3%) using BMI.

**Fig. 1:**
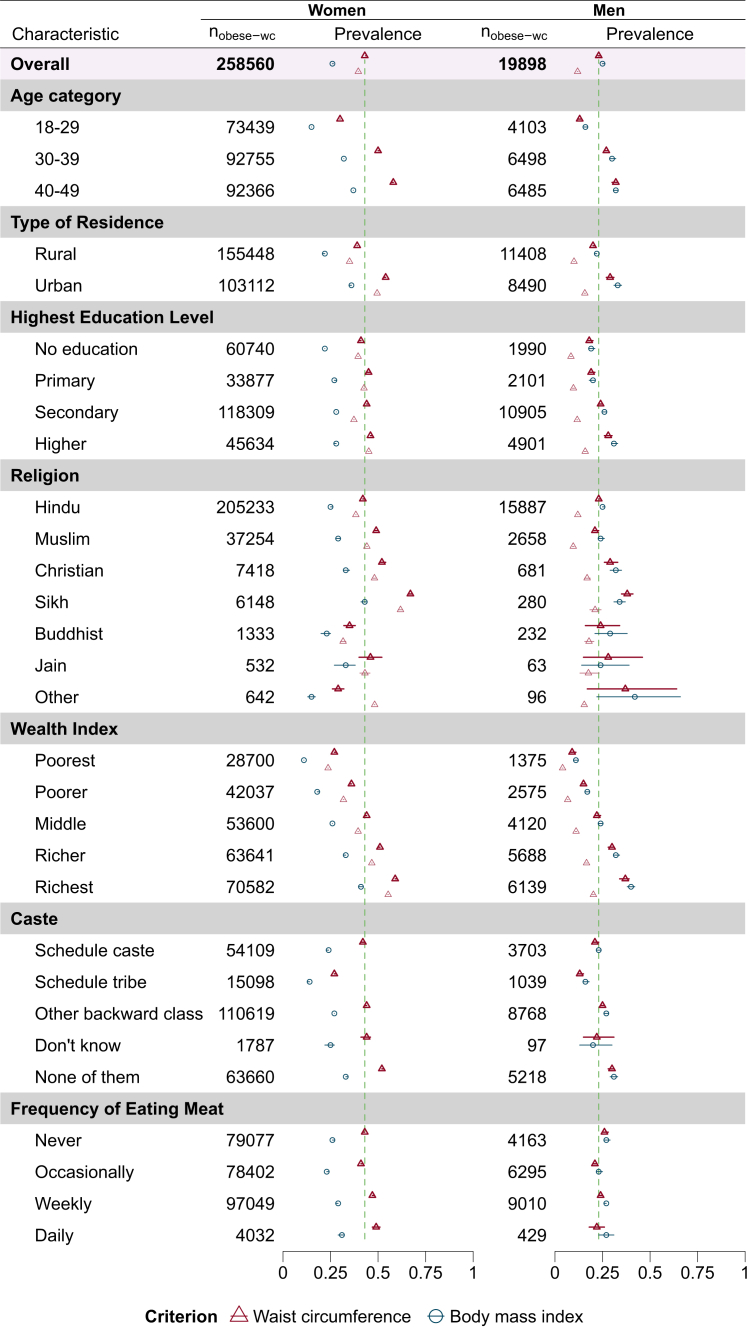
Prevalence estimates for obesity among men and women in the sample population based on waist circumference and body mass index. n_obese-wc_: number of obese individuals based on waist circumference cut-off. The estimates for obesity using waist circumference cut-off in lighter shade represents estimates from the original paper and only presented for variables where categories are same. Obesity is defined for women and men by a waist circumference of ≥80 cm and ≥90 cm, and a BMI of ≥25 kg/m^2^ for both, respectively, according to “The Asia–Pacific Perspective: Redefining Obesity and Its Treatment, 2000.” Prevalence estimates are presented as point estimates with 95% confidence intervals. The dashed vertical reference line indicated the prevalence of obesity based on the waist circumference cut-off. The model includes 594,489 women and 86,347 men in the sample population. For men, the highest age category is 40–54 years, which is 40–49 years for women. The Other religion category includes Parsis or Zoroastrians, Jews, and those of no religion.

The obesity estimates using the waist circumference cutoff were consistently higher than those using the BMI cutoff amongst women—a phase shift—but similar for men. In the multivariable model, age category and wealth index showed the largest and most consistent effect for both sexes, with higher odds for older and richer individuals (Supplementary Figure S2). Southern and northern states like Kerala, Andhra Pradesh, Punjab, Haryana, and Delhi exhibit higher abdominal obesity prevalence, while central and eastern states like Madhya Pradesh, Jharkhand, Nagaland, and Meghalaya report lower prevalence. Further, as shown in the district estimates for women, there is heterogeneity within states (Supplementary Figures S3 and S4).

In comparing our findings to the original paper,^7^ several discrepancies became evident. There are arbitrary methodological choices like not using a contemporary criteria for defining obesity, combining levels in the ‘frequency of eating meat’ variable (never and occasionally combined into “Vegetarian/Occasionally non-vegetarian”; weekly and daily to “Non-vegetarian”), excluding outliers beyond ± 3 SD.^11^ Unlike our analysis, the prevalence estimates show large differences for men. While the direction of the adjusted odds ratio remained the same, the point estimates differed. Last, given the large sample size, the model could have included other relevant variables like alcohol consumption that have been used by previous papers.^12^

Reproducibility is a cornerstone of science.^13^ We offer three suggestions to make it a reality. First, the journal needs to articulate policies on research reproducibility while engaging with the authors and readers to incorporate their views.^14^ Second, create meaningful incentives for researchers to share their code and data, for example, by inviting reproducibility studies.^15^^,^^16^ Third, make code sharing a mandatory requirement for publishing articles using publicly available data. Transitioning from an ideal to a tangible reality where reproducible research is the norm is a necessity because only open science is good science.

## Contributors

TG conceptualised the idea. Both TG and AG worked collaboratively on the formal analysis and manuscript preparation.

## Data sharing statement

The analysis code is available through the public GitHub repository at https://github.com/apg1997/NFHS_AbdominalObesity. The National Family Health Survey 2019–2021 data can be requested from the Demographic and Health Surveys (DHS) Program at https://dhsprogram.com/.

## Editor note

The Lancet Group takes a neutral position with respect to territorial claims in published maps and institutional affiliations.

## Declaration of interests

We have no conflicts of interests to declare.
