# Supplementary material for: Prerequisite for reproducible science: a call to embrace code sharing
Source: Lancet Reg Health Southeast Asia. 2024 Aug 23;29:100472. doi: 10.1016/j.lansea.2024.100472 (PMC11386511; doi:10.1016/j.lansea.2024.100472)
Supplement: Supplementary information [file mmc1.docx]

**SUPPLEMENTARY INFORMATION**

**Prerequisite for reproducible science: a call to embrace code sharing**

*Arkaprabha Gun^a#^, Tushar Garg^b,c#^**

^a^Department of Infectious Disease Epidemiology and Dynamics, London School of Hygiene & Tropical Medicine, London, UK
^b^Johns Hopkins India, Lucknow, Uttar Pradesh, India
^c^Department of International Health, Johns Hopkins Bloomberg School of Public Health, Baltimore, USA

^#^Both authors contributed equally to this work.

*Corresponding author

Johns Hopkins India, 202, Ratan Square, Vidhan Sabha Road, Lucknow, Uttar Pradesh, India 226001

*E-mail address*: [tgarg2@jhu.edu](mailto:tgarg2@jhu.edu) (T. Garg)

**Supplementary Figure 1. Sample selection flowchart**

Note: The exclusion steps are sequential in the order they appear.

**Supplementary Figure 2.** Model estimates from the univariate and multivariate logistic regression model showing association of abdominal obesity with socioeconomic factors in the women and men sample population


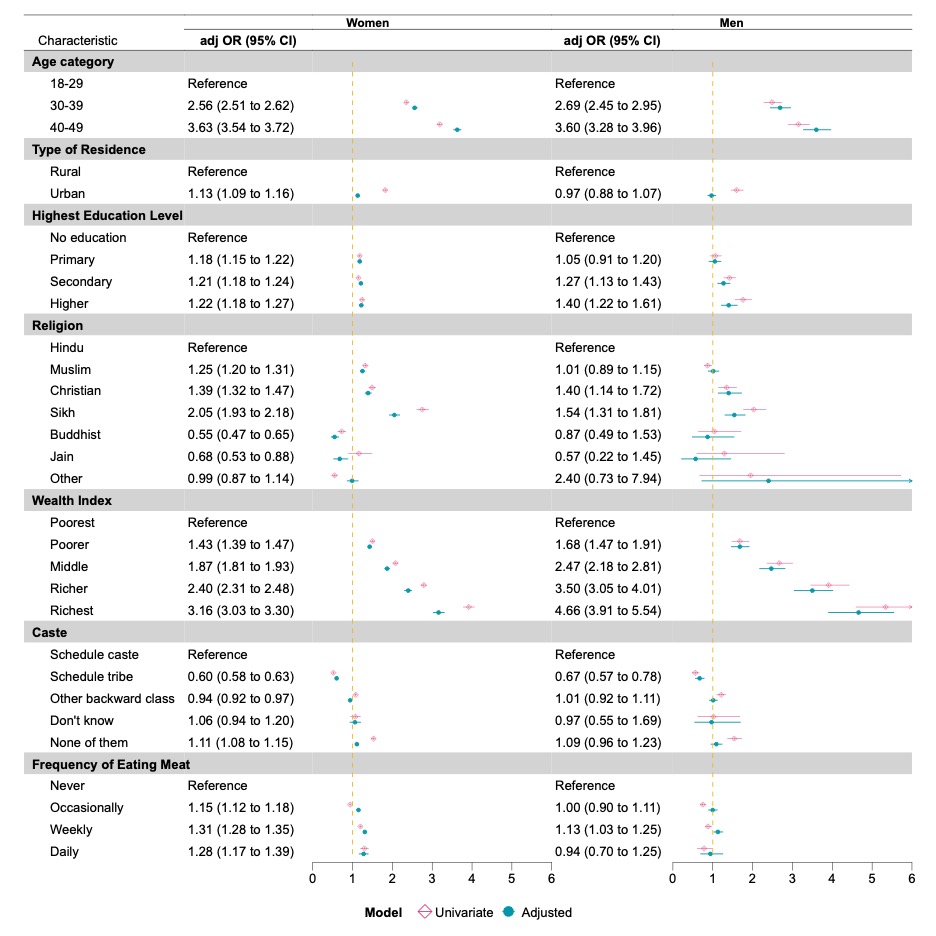


**Note:**adj OR: adjusted odds ratio for the multivariate or adjusted mode; CI: Confidence Interval

The model includes 594,489 women and 86,347 men in the sample population. For men, the highest age category is 40-54 years, which is 40-49 years for women. The Other religion category includes Parsis or Zoroastrians, Jews, and those of no religion.

**Supplementary Figure 3.** State-level abdominal obesity estimates among men and women in the sample population.

**Note:** The model includes 594,489 women and 86,347 men in the sample population.

**Supplementary Figure 4.** District-level abdominal obesity estimates among women in the sample population.

**Note:**The model includes 594,489 women and 86,347 men in the sample population. The district-level representative estimates are not available for men because of the smaller sample size compared to the women's sample population.
